# Male and female syringeal muscles exhibit superfast shortening velocities in zebra finches

**DOI:** 10.1242/jeb.246330

**Published:** 2024-04-08

**Authors:** Nicholas W. Gladman, Coen P. H. Elemans

**Affiliations:** Vocal Neuromechanics Lab, Sound Communication and Behaviour Group, Department of Biology, University of Southern Denmark, 5230 Odense M, Denmark

**Keywords:** Muscle performance, Isovelocity, *V*_max_, Vocal performance, Song learning

## Abstract

Vocalisations play a key role in the communication behaviour of many vertebrates. Vocal production requires extremely precise motor control, which is executed by superfast vocal muscles that can operate at cycle frequencies over 100 Hz and up to 250 Hz. The mechanical performance of these muscles has been quantified with isometric performance and the workloop technique, but owing to methodological limitations we lack a key muscle property characterising muscle performance, the force–velocity relationship. Here, we quantified the force–velocity relationship in zebra finch superfast syringeal muscles using the isovelocity technique and tested whether the maximal shortening velocity is different between males and females. We show that syringeal muscles exhibit high maximal shortening velocities of 25*L*_0_ s^−1^ at 30°C. Using *Q*_10_-based extrapolation, we estimate they can reach 37–42*L*_0_ s^−1^ on average at body temperature, exceeding other vocal and non-avian skeletal muscles. The increased speed does not adequately compensate for reduced force, which results in low power output. This further highlights the importance of high-frequency operation in these muscles. Furthermore, we show that isometric properties positively correlate with maximal shortening velocities. Although male and female muscles differ in isometric force development rates, maximal shortening velocity is not sex dependent. We also show that cyclical methods to measure force–length properties used in laryngeal studies give the same result as conventional stepwise methodologies, suggesting either approach is appropriate. We argue that vocal behaviour may be affected by the high thermal dependence of superfast vocal muscle performance.

## Teaser

**Summary:** Zebra finch vocal muscle exhibits superfast shortening velocities that correlate with isometric performance. The low power output highlights the importance of high-frequency operation in muscles modulating vocal output.

## INTRODUCTION

Vocal communication is of key importance to songbirds, with vocalisations playing important roles in behaviours ranging from predator avoidance to conspecific recognition and mate choice (Bradbury and Vehrencamp, 2011; Kelley et al., 2008; Nowicki and Searcy, 2004). Vocal signalling demands a high degree of motor control precision, and the premotor circuitry in songbirds and mammals indeed has millisecond precision (Chi and Margoliash, 2001; Nieder and Mooney, 2020; Pomberger et al., 2018). The precise execution of descending motor control is facilitated by the vocal muscles of the mammalian larynx and avian syrinx that, by modifying vocal fold tension and positioning, control key acoustic parameters such as amplitude and fundamental frequency (Riede and Goller, 2010). In both the larynx and syrinx, superfast muscles have evolved that are optimised for speed (Elemans et al., 2008, 2011, 2004). Superfast muscles produce positive work at ≥100 cycles s^−1^ and those in the larynx and syrinx can modulate vocal features up to 200–250 Hz (Elemans et al., 2008, 2011; Mead et al., 2017). To achieve such remarkable performance, superfast muscles exhibit several cellular and molecular adaptations, such as large volume proportions of sarcoplasmic reticula, amplified expression of calcium-handling systems, increased fibre diameter, as well as expressing unique myosin isoforms (Hoh, 2005; Mead et al., 2017; Rome, 2006; Rome and Lindstedt, 1998), that allow them to operate at the maximum operational speed set by fundamental constraints in synchronous muscle architecture (Mead et al., 2017). However, these adaptations for speed come at the expense of force, because of reduced myofibrillar area and lower numbers of formed cross-bridges (Rome and Lindstedt, 1998; Rome et al., 1996). Superfast muscles evolved independently in sound-producing organs in ray-finned fish, birds and mammals, suggesting paramount importance in vocal communication (Mead et al., 2017).

The mechanical performance of muscle depends on both its force–length and force–velocity relationships. The assessment of the mechanical properties in avian vocal muscles has so far largely focused on isometric (Adam et al., 2023) and cyclic performance (Elemans et al., 2008, 2006), but the force–velocity relationship remains unknown. Force–velocity properties in vocal systems have, to date, been measured in mammalian and amphibian laryngeal muscles, such as the thyroarytenoid (TA) muscle of the baboon (*Papio* sp.; Mardini et al., 1987), rat (*Rattus norvegicus*) TA muscle (McMullen and Andrade, 2006), human (*Homo sapiens*) TA and cricoarytenoid (CA) muscles (D'Antona et al., 2002; Sciote et al., 2002), dog (*Canis familiaris*) TA, CA and transverse arytenoid (AT) muscles (Alipour and Titze, 1999; Toniolo et al., 2007), and in the tensor chordarum, external and internal oblique trunk muscles of the hylid frogs (*Hyla* spp.) (Girgenrath and Marsh, 1999, 2003; McLister et al., 1995). However, these muscles are all slower than avian superfast syrinx muscles. Although force–velocity properties have previously been measured in invertebrate superfast muscles, such as insect flight (Fitzhugh and Marden, 1997; Josephson, 1984; Malamud and Josephson, 1991; Marden, 1995) and leg muscles (Ahn and Full, 2002; Eldred et al., 2010), we currently lack quantification of the force–velocity relationship in any terrestrial vertebrate superfast muscle (but see Rome et al., 1996). Thereby we lack insight in a critical feature describing the mechanical performance of superfast muscles.

Here, we measured force–velocity relationships in a vertebrate superfast muscle, the syringeal muscles of the zebra finch (*Taeniopygia guttata*). The songbird vocal system is sexually dimorphic in the zebra finch. Males use song to attract mates and enhance pair-bonding (Griffith, 2019; Loning et al., 2022), whereas females do not produce song. Females have considerably smaller syringeal muscles than males (Christensen et al., 2017; Wade and Buhlman, 2000). Alongside these anatomical differences, functional differences of the muscles themselves are also present. The twitch parameters of male syringeal muscles are nearly twice as fast as female syringeal muscles (Adam and Elemans, 2020; Elemans et al., 2008), and express more myosin heavy-chain gene MYH13 with superfast properties (Mead et al., 2017). We hypothesise that faster twitch and tetanus parameters, such as twitch and tetanic rise times, will correlate with faster shortening velocities. However, we currently lack non-isometric speed measures in male and female muscles.

To further enhance our understanding of vocal muscle performance in a comparative context, we also compared two methodologies of assessing force–length profiles. To determine force–length properties in laryngeal muscles, Alipore-Haghighi et al. (1991) applied cyclical length changes at 1 Hz both passively and during electrical stimulation. However, such long duration stimulations do not allow the muscle to relax between successive stimuli, which likely impacts cross-bridge formation, breaking, as well as causing residual passive force enhancement owing to the action of titin and other non-contractile elements (Herzog, 2019). In addition, these long stimulations potentially deplete the energy storage and fatigue the muscle and therefore are not routinely used in conventional muscle physiology. Therefore, we also tested whether this cyclical long duration stimulation methodology results in similar force–length profiles and optimal length (*L*_0_) compared with commonly used iterative stepwise approaches.

## MATERIALS AND METHODS

### Bird housing conditions

Adult zebra finches [*Taeniopygia guttata* (Vieillot 1817)] were housed within an aviary (2.48×2.36×2.03 m; length×width×height) at the University of Southern Denmark. Animals were kept in a mixed-sex group of approximately 70 animals, on a 13 h:11 h light:dark cycle at 20±3°C. Food, water and cuttlebones (*Sepia* spp.) were available to all animals *ad libitum* within the aviary. We used 9 female and 8 male zebra finches; at the time of use, females were 266±160 days old and males were 339±142 days old.

### Muscle fascicle preparation and mounting

Zebra finches were euthanised via an overdose of isoflurane (Attane vet, ScanVet Animal Health A/S, Fredensborg, Denmark). No specific ethical approval was required for subsequent experiments in accordance with Danish law, as approved by the Danish Animal Experiments Inspectorate (Copenhagen, Denmark).

The syrinx was extracted through a vertical incision along the sternum; during the dissection process, the chest cavity was regularly flushed with ice-cold oxygenated avian buffer solution (see Table S1 for composition; Adam et al., 2021). The extracted syrinx was placed in 1±1°C oxygenated avian buffer solution within a Petri dish, temperature was maintained using an aluminium cooling plate with constant circulation through a chiller (Lauda RM6 Refrigerated Circulating Bath, Lauda Dr. R. Wobser GMBH & Co., Lauda, Germany).

We focused on the dorsal tracheobronchial (DTB) muscle, from which we have the most information on isometric contractile performance (Adam and Elemans, 2019, 2020; Adam et al., 2021, 2023; Mead et al., 2017). The DTB muscle was dissected out of the extracted syrinx. The side from which muscle was dissected out was randomly determined to minimise left–right biases (10/17 59% from left, 7/17 41% from right). A small piece of bone (bronchial half-ring B2) and associated connective tissue (clavicular air sac membrane, CASM) were kept at either end of the muscle. We securely fastened both ends into aluminium foil T-clips (Photofabrication Ltd, St Neots, Cambs, UK). Clips were used to connect the muscle to 100 µm diameter stainless steel hooks made from Austerlitz insect pins (Minutiens 0.1 mm, Entomoravia, Slavkov u Brna, The Czech Republic) connected to a force-transducer (400A, Aurora Scientific, Aurora, ON, Canada) at one end, and a high-speed length controller (322C, Aurora Scientific) at the other. To increase the resonance frequency and thus the response speed of the force transducer, we reduced the weight of the load sensing cell as much as possible. Therefore, we used shortened glass tubes, the lowest possible amount of wax to connect short stainless-steel rods and lightweight aluminium clips. Together, this resulted in a resonance frequency of approximately 0.5 kHz.

Both the length controller and the force transducer were mounted on 3D micro-manipulators. The muscle was held within a temperature-controlled bath (cooled via an 826A system, Aurora Scientific) and continually superfused with avian buffer solution using a peristaltic pump (Ole Dich Instrumentmakers ApS, Hvidovre, Denmark). Platinum electrodes ran parallel to the muscle on both sides and were used for electrical stimulation of the muscle via a high-power, bi-phase stimulator (701C Aurora Scientific). Signals were low-pass filtered (10 kHz, inline filter, Thor Labs, Newton, NJ, USA) and digitised at 40 kHz, 16 bits (PCI 6259, National Instruments, Austin, TX, USA). All control software was written in MATLAB (2021b, MathWorks, Natick, MA, USA).

### Protocol

At a temperature of 30±0.5°C, slack muscle length was measured using digital callipers, and adjusted to 110% of this initial length using a micromanipulator. Initial slack length was set based on prior experience. The stimulation conditions were optimised for tetanic force using a fixed voltage (80 V) and modifying the current amplitude and duration of the stimulus. Next, we increased the stimulation frequency from 100 to 500 Hz in 50 Hz steps to find the tetanic frequency where isometric force was maximal.

#### Force–length curve

We next determined the optimal length (*L*_0_) of muscle by applying a series of isometric tetani at different lengths. This began at 110% slack length, with length modified in steps of 0.25 mm. Modification of muscle length began by increasing the length from the initial length. If muscle force declined, the muscle length was decreased from the initial length. The length at which maximal force was recorded was defined as *L*_0_. Between each successive tetanus, the muscle was allowed to rest for 3 min.

After length optimisation, the muscle was stimulated to produce an isometric twitch and tetanus at *L*_0_.

#### Force–velocity curves

To measure force–velocity properties of syringeal muscle, we used the isovelocity technique. This technique involves controlling muscle shortening velocity rather than force, where the muscle is shortened at set velocities and the resulting force is measured. Preliminary tests using other methodologies (slack tests and force-holds) proved ineffective, because muscular force would recover too rapidly during slack test measures, and muscle force was too low to reliably use force-holds in a dual-mode lever system.

**Fig. 1.**
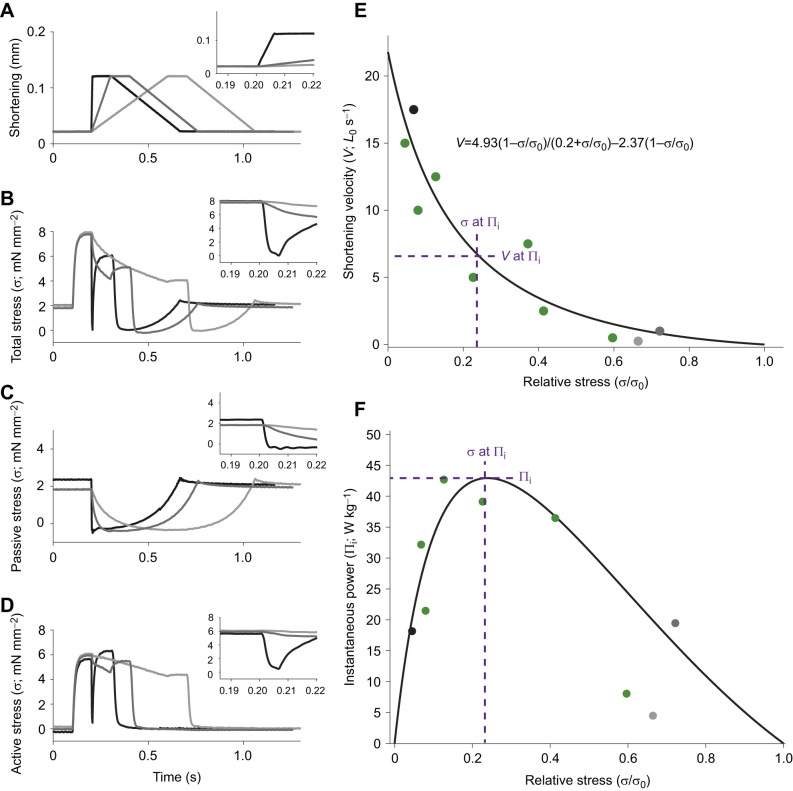
**Force–velocity protocol and analysis example.** (A) Length changes at different ramp velocities. (B) Total muscular stress when stimulated, where the muscle tetanised before being shortened at the differing ramp velocities. (C) Passive stress during shortening. (D) Active stress, calculated as total stress (B) minus passive stress (C). Insets in A–D show a zoomed version of the ramp process. Graded shades of grey are used to represent different ramp velocities. (E) Resulting force–velocity profile including the data shown in A–D using points of corresponding colours; green points show data not included in A–D. (F) Power output derived from the force–velocity profile; powers calculated from shown data are displayed using points of corresponding colours to lines shown in A–D; green points indicate data not shown. Purple lines in E and F are used to highlight the maximum shortening velocity (*V*_max_), stress at peak power (σ at Π_i_), velocity at peak power (*V* at Π_i_) and peak power (Π_i_). Data shown are left DTB preparation from female GW423 at 30°C using velocity-capped protocol 1 (no-step).

During isovelocity measurements, we set the length to 110% *L*_0_, and tetanised the muscle (Fig. 1). While stimulating, we shortened the muscle at constant velocity back to *L*_0_. Once stimulation stopped, the muscle was gradually returned to 110% *L*_0_ at 0.3*L*_0_ s^−1^. This procedure (protocol 1) was repeated for a range of isovelocity values from 0.25 to 35*L*_0_ s^−1^ in series from the fastest value down to the slowest to minimise muscle fatigue. As this process may have caused an order effect, three of the preparations were ran in a randomised order, these measures did not differ from previous measures, suggesting any order effects may be minor. To correct for passive force as well as the impacts of serial elasticity, we performed two runs per isovelocity value: one with stimulation (active) and one without stimulation (passive). The muscle was given 3 min to rest between successive active isovelocity runs.

In addition to these tests, three of the muscles were also used to test the protocol of Claflin and Faulkner (1989) that adds a small transient shortening just before the isovelocity ramp (protocol 2; Fig. S1). This step aids the muscle in achieving steady-state force production during the isovelocity ramp. Unlike in protocol 1, initial muscle length in protocol 2 was set to 105% *L*_0_ and shortened to 95% *L*_0_ (following Claflin and Faulkner, 1989). To test for residual force depression (Herzog and Leonard, 2007), we also extended the tetanic stimulation beyond the end of the isovelocity manoeuvre in these three muscles.

Preliminary tests at 40°C in syringeal muscle were hampered by rapid recovery of muscular force, as seen in previous slack tests. Therefore, subsequent experiments were carried out at 20 and 30°C. Introducing the shortening step helped to achieve steady-state at 20 and 30°C but also introduced an oscillatory force transient that increased with step size. At an increased temperature of 40°C, the higher shortening velocities would likely require larger step sizes (Claflin and Faulkner, 1989), which would cause ringing of the force trace that would last longer than the isovelocity curve. Furthermore, the step size was limited to the 10% of *L*_0_, and at the high speeds at 40°C, larger steps would be needed. Lastly, fast shortening needed at 40°C was limited by the speed of the motor of the high-speed length controller. Because of these compound effects, force–velocity curves were determined at two temperatures – 20 and 30°C – where maximum shortening velocity could be measured accurately.

#### Comparison of force–length curves obtained via stepwise and cyclic methodologies

Next, we tested whether stepwise and continuous cyclical protocols result in different force–length curves. To ensure muscle was not damaged during sustained stimulation, cyclic methods were carried out after force–velocity measurements. At *L*_0_ and 30°C, muscle fascicles were subjected to two 1 Hz sinusoidal length changes (±0.5 mm from *L*_0_). This consisted of one unstimulated (passive) length change, and one stimulated (active) length change (following Alipour–Haghighi et al., 1991).

### Cross-sectional area of preparation

Lastly, we determined the length and cross-sectional area (CSA) of the preparation. We placed a 5 mm silver right angle prism mirror (MRA05-P01, Thor Labs) next to the preparation in the bath and took an image including the top and sideview using a camera mounted on a fluorescent stereo microscope (Flexacam C3 on a M165 FC, Leica Microsystems, Heerbrugg, Switzerland). From this image, we measured muscle width (*W*_m_) and depth (*D*_m_) at three points along the muscle fibre bundle using ImageJ v1.53 (National Institutes of Health, Bethesda, MD, USA). These three points were used to calculate the average width and depth of the muscle. From these, we calculated CSA assuming an ellipsoid cross-section: 
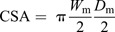
.

### Data analysis

All data were analysed using MATLAB (2021b, MathWorks).

#### Force–velocity

The force–velocity relationship of a muscle is described by the hyperbolic-linear equation (Marsh and Bennett, 1986):
(1)

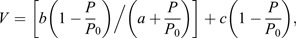
where *a*, *b* and *c* represent constants, *P* is force and *P*_0_ is peak isometric force. Peak isometric force (*P*_0_) was the mean peak force during the tetanus preceding ramp measurements. Force (*P*) was the force at the end of the ramp (Fig. 1). Forces were converted to stresses by dividing by the CSA. We determined *V*_max_ by extrapolating the hyperbolic-linear fit to zero force.

#### Full versus velocity-capped force–velocity curves

The data obtained using protocol 2 showed muscle could achieve a steady-state up to 17.5*L*_0_ s^−1^. Therefore, we tested whether using a velocity cap of 17.5*L*_0_ s^−1^ on our dataset obtained using protocol 1 impacted the results. These comparisons showed velocity-capped and non-capped datasets did not return different force–velocity profiles (in terms of *V*_max_ and curvature). Therefore, all subsequent data are presented using this velocity cap.

#### Power output

The power output of muscle was estimated by calculating the product of force×velocity from the force–velocity curves. To quantify the degree of curvature of force–velocity curves, we calculated the power ratio as Π_i_/(σ_0_×*V*_max_), where Π_i_ is the peak instantaneous power. Power ratios range from 0 to 1, with lower values indicating a higher degree of curvature.

#### Performance extrapolation to body temperature

Unfortunately, we were unable to effectively measure force–velocity at physiological body temperature of 39–41°C (Ellerby and Askew, 2007) owing to the muscle generating force too rapidly for the high-speed length controller. Therefore, we instead measured at 20 and 30°C, and extrapolated to a physiological temperature of 40°C using *Q*_10_ values:
(2)

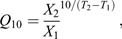
where *X*_2_ is velocity or other parameter at the higher temperature (*T*_2_) and *X*_1_ is the same parameter at the lower temperature (*T*_1_). We used the velocity-capped dataset (protocol 1) for these calculations. Applying the *Q*_10_ value measured between 20 and 30°C to estimate parameter values at 40°C likely overestimates performance. Therefore, we used these values to give an upper range estimate of the maximum shortening velocity at a physiological temperature of 40°C. For lower range estimates, we used the minimum value of 1.50 as reviewed by Bennett (1984) across vertebrate muscle types.

#### Force–length

**Fig. 2.**
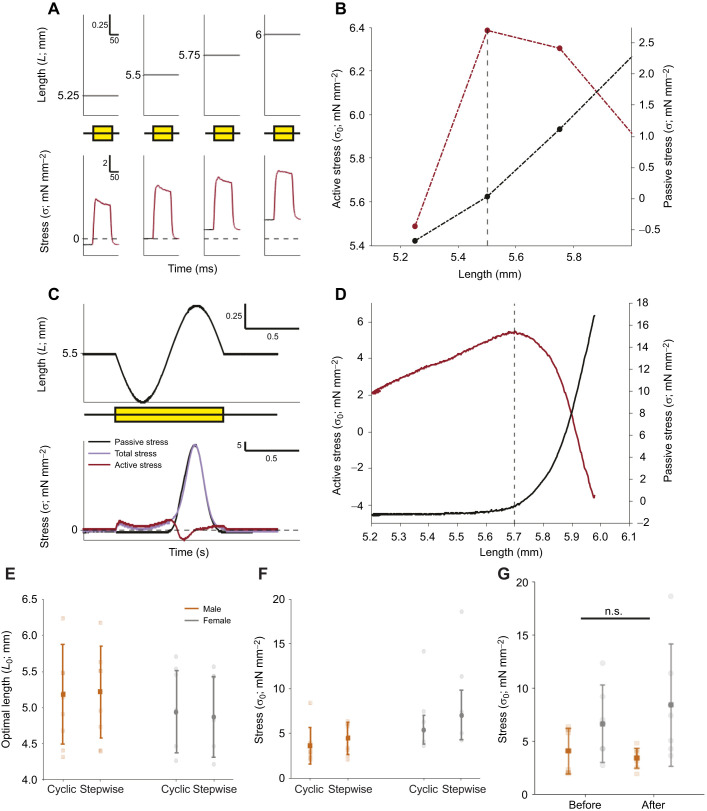
**Cyclic and stepwise protocols provide the same optimal length and stress in vocal muscles.** (A) Stepwise approach muscle length is increased in steps and tetanised at each length. (B) Resulting profile from this approach with active force shown in red and passive in black, vertical dashed line shows the optimal length (*L*_0_). (C) Cyclic approach, muscle is stimulated throughout a 1 Hz length change (±0.5 mm from starting length), top panel shows length change, and bottom shows resulting stress outputs. Black line shows the passive stress, purple total stress and red active stress (calculated as total stress minus passive stress). (D) Resulting profile from approach in C; active stress shown in red and passive in black, the vertical dashed line indicates *L*_0_. Yellow boxes in A and C are used to show the stimulation period. (E) *L*_0_ from the stepwise and cyclic methodologies. Solid filled orange squares (male; *N*=8) and grey circles (female; *N*=6) show the mean±s.d., translucent squares and circles show raw data. (F) The stress (σ_0_) at the optimal length during stepwise and cyclic methodologies. Solid filled orange squares (male) and grey circles (female) show the mean±s.d., translucent squares and circles show raw data. (G) Isometric stress before and after being stimulated for 1 s. Stress 5 min after prolonged stimulation did not significantly differ from previous tetani. Male data (*N*=8) are shown in orange, and female (*N*=6) in grey, solid colours show the mean±s.d., translucent show individual data points.

At each length step, the active and passive force was obtained and used to plot force–length profiles (Fig. 2). In the stepwise approach, passive force was the average force during the first 100 ms prior to stimulation. Active force was calculated as the average force over the plateau region of the tetanus minus the passive force. The force plateau was detected as the region between the first instance of the maximum value and the point at which force changes significantly (findchangepts function). These points were visually assessed to ensure only the plateau region was sampled.

The cyclic approach used the unstimulated lengthening–shortening cycle as the passive force (Fig. 2). Active force was calculated by subtracting the passive force from the stimulated force trace at each time point. To ensure data were aligned, the start- and end-points were set and detected using the find function in MATLAB. All forces (*P*) were converted to stresses (σ) by dividing by the muscle CSA.

#### Isometric contractile performance

To assess whether previous measures of isometric contractile function are related to non-isometric properties we assessed four measures of isometric performance: time to peak twitch force (*t_P_*_tw_), time to peak tetanus force (*t_P_*_0_), full-width of the twitch at half force (FWHM) and time from peak twitch force to half of this force (twitch half relaxation time, RT_50_). All isometric contractile performance measures were assessed at *L*_0_ and 30°C.

#### Statistical analysis

Statistical testing was conducted using IBM SPSS Statistics 28 (IBM, Armonk, NY, USA). Data are presented as means±s.d. throughout. All data were tested for normality (Shapiro–Wilk test) and homogeneity (Levene's test) prior to statistical testing. Data that were non-normally distributed were transformed to meet these assumptions via log transformation. A critical *P*-value of 0.05 was used to indicate significant differences. Male–female comparisons were made using independent samples *t*-tests. Comparisons of force–length methodologies were made using paired *t*-tests. Correlations between isometric contractile dynamics and shortening velocities were made using Pearson's correlation coefficient.

## RESULTS

### Force–velocity relationship of syringeal muscle is not sex dependent

**Fig. 3.**
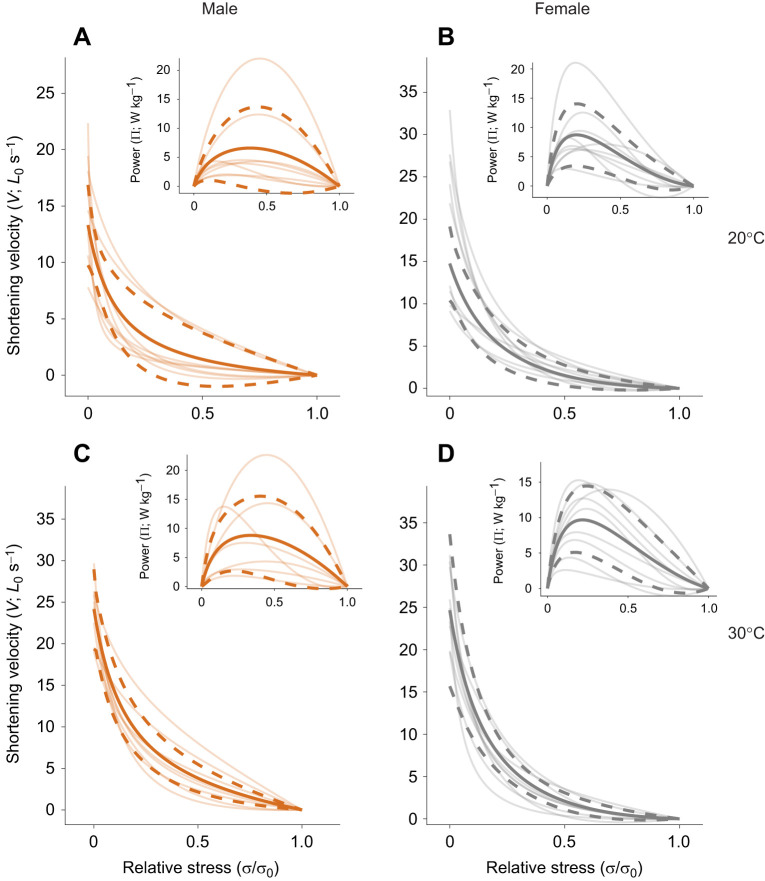
**Force–velocity and force–power relationships in zebra finch syrinx muscle.** (A,C) Force–velocity curves of male (orange; *N*=8) dorsal tracheobronchial (DTB) muscle at (A) 20°C and (C) 30°C. (B,D) Force–velocity curves of female (grey; *N*=9) DTB muscle at (B) 20°C and (D) 30°C. Insets show the resulting force–power relationships, calculated as force×velocity. The mean (solid) and standard deviation (dashed) are thick lines, whereas individual curves are translucent. All shown data was obtained using velocity-capped protocol 1 (no-step).

We hypothesised that male–female differences in isometric performance would also be present in the non-isometric properties of muscle function. We measured force–velocity curves in superfast zebra finch syringeal muscles using the isovelocity protocol and found that greater muscular force is associated with decreased velocities, typical for skeletal muscle (Figs 1 and 3).

The DTB muscle preparations did not always achieve a steady-state, i.e. a constant force during the isovelocity manoeuvre. In a subset of three female DTB muscles, we applied a small shortening transient preceding the isovelocity ramp (Figs S1, protocol 2) to aid the muscle in achieving steady-state force production during the isovelocity ramp (Claflin and Faulkner, 1989). This approach did not return significantly different estimates of maximum shortening velocity (*t*=1.4, d.f.=2, *P*=0.3, *N*=3). As muscle preparations in this subset did reach a steady-state up to 17.5*L*_0_ s^−1^, we next capped the entire dataset in our analysis to include only data to a maximum of 17.5*L*_0_ s^−1^. The addition of this velocity cap did not return significantly different *V*_max_ (*t*=0.06, d.f.=15, *P*=0.95) or power ratios (*t*=0.32, d.f.=15, *P*=0.75; Fig. S2). All data presented here are therefore velocity-capped to 17.5*L*_0_ s^−1^.

Previous work has found male–female differences in the twitch dynamics and muscle fibre types of zebra finches. Here, we also found twitch force development rates differed between sexes; however, non-isometric parameters did not differ significantly between sexes (Table 1, Fig. 3). We found the velocity at maximum power, relative stress at maximum power and power ratios were not different between male and female muscles at either of the two temperatures measured. Taken together, these findings indicate that force–velocity curves do not differ significantly between male or female muscle at 20 or 30°C, and so we pooled the data. At 30°C, the peak shortening velocity is 24.79±4.02*L*_0_ s^−1^ (*N*=17), with a power output of 13.40±7.22 mW g^−1^. This power output was achieved at 29±8% of *V*_max_ and 31±6% of σ_0_. The power ratio of force–velocity curves at 30°C was 0.11±0.07, indicating a low degree of curvature.

**Table 1.**
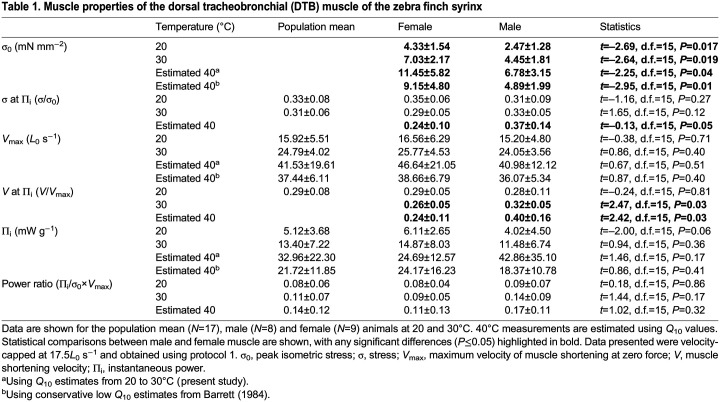
Muscle properties of the dorsal tracheobronchial (DTB) muscle of the zebra finch syrinx

We continued the tetanic stimulation of the muscle beyond the isovelocity manoeuvre to assess whether residual force depression was taking place in both protocols 1 and 2 using a subset of three female DTB muscles. We found that the force immediately following the isovelocity manoeuvre was approximately 80% of the tetanic force preceding the manoeuvre. The muscle length after the ramp was 5% below the optimum in these experiments, and based on our force–length measurements, force should be approximately 80% of the tetanic force at 95% *L*_0_ (see Fig. 2). Because these values are similar, we conclude that residual force depression was not occurring.

The influence of temperature on DTB muscle performance was similar in both male and female muscle (Tables 1 and 2). However, male muscle produced significantly lower stress at both 20 and 30°C (Table 1).

**Table 2.**
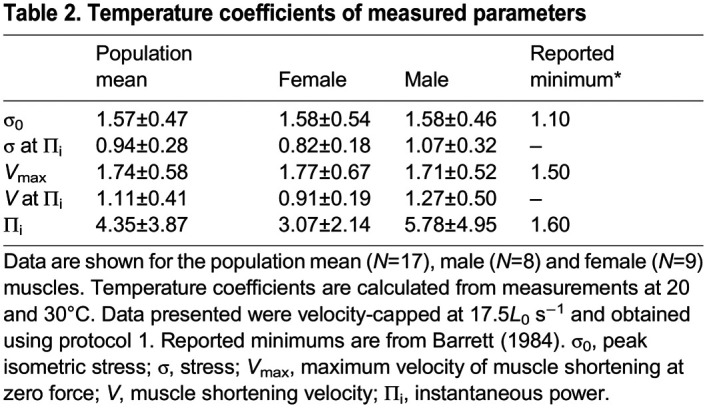
Temperature coefficients of measured parameters

**Fig. 4.**
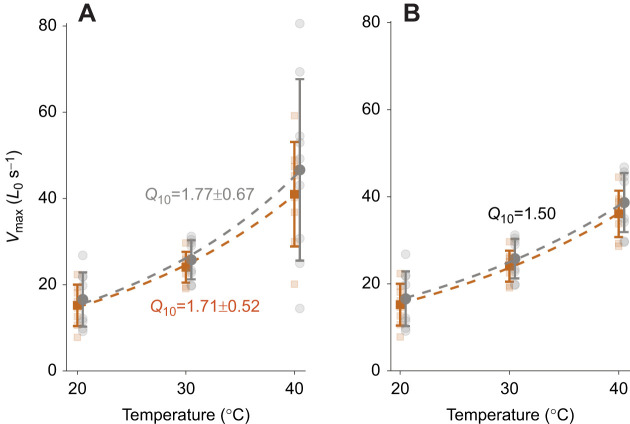
**Maximum shortening velocity extrapolation of DTB muscle to body temperature.** (A) Shortening velocity at 40°C estimated using *Q*_10_ values calculated from measurements at 20 and 30°C in male (orange; *N*=8) and female (grey; *N*=9) DTB muscle, and (B) assuming a conservative *Q*_10_ value of 1.50. Means±s.d. are shown as filled squares and circles; raw data are shown as translucent. The mean±s.d. *Q*_10_ temperature coefficients are shown for each sex. Male and female data are slightly offset in temperature for clarity. All shown data were obtained using velocity-capped protocol 1 (no-step).

We used both the *Q*_10_ values estimated from measures taken at 20 and 30°C, and a conservative lower level of *Q*_10_ values reported in locomotory muscles (Table 2), to extrapolate DTB function to a body temperature of 40°C. From these measurements, we estimated *V*_max_ to be between 37.44±6.11 and 41.53±19.61*L*_0_ s^−1^ (Fig. 4), and power output to be between 21.72±11.85 and 32.96±22.30 mW g^−1^ at 40°C.

### Relationship between isometric contractile dynamics and shortening velocity

**Fig. 5.**
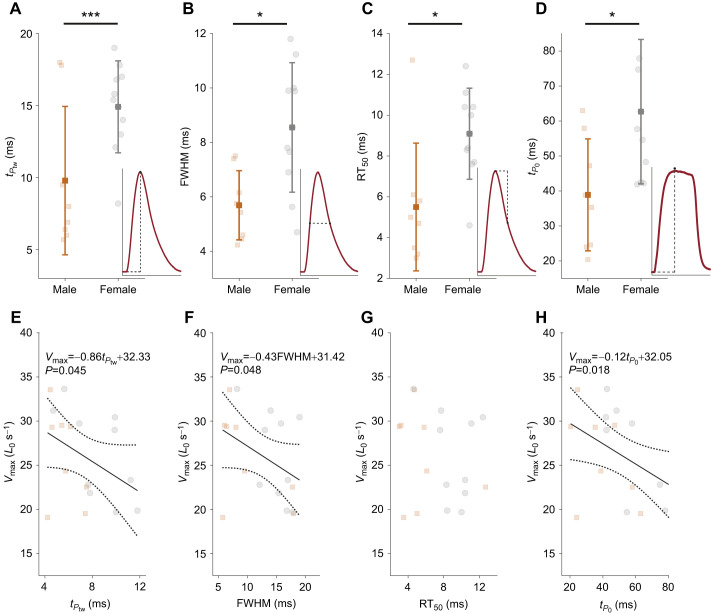
**Isometric properties that predict muscle shortening velocity.** Isometric contractile features of DTB muscle at 30°C: (A) time to peak twitch (*t_P_*_tw_), (B) full width at half maximal force time (FWHM), (C) twitch half relaxation time (RT_50_) and (D) time to peak tetanus (*t_P_*_0_). Insets in A–D give examples of how these parameters were measured. (E–H) The relationship between *V*_max_ and (E) *t_P_*_tw_, (F) FWHM, (G) RT_50_ and (H) *t_P_*_0_. Correlation equations shown in E, F and H were all significant, with respective *P*-values of 0.045, 0.048 and 0.018. Data from males (*N*=8) are shown in orange, and females (*N*=9) in grey, translucent colours show individual datapoints, solid colours show the means±s.d., asterisks indicate significant differences (*P≤0.05 and ***P≤0.001). All *V*_max_ values were obtained using velocity-capped protocol 1 (no-step).

The twitches of male muscles were significantly faster than those of female muscles (Fig. 5), with full width at half maximal force (FWHM) times of 9.79±5.15 ms in males (*N*=8) and 14.91±3.19 ms in females (*N*=9) at 30°C (*t=*−2.59, d.f.=15, *P*=0.02). Other aspects of twitch performance, such as the time to peak twitch (*t_P_*_tw_) and twitch half relaxation time (RT_50_), were also significantly faster in male than in female muscle at 30°C: *t_P_*_tw_ was 5.69±1.27 ms in males and 8.95±1.96 ms in females (*t=*−4.19, d.f.=15, *P*=<0.001), and RT_50_ was 5.50±3.13 ms in males and 9.09±2.23 ms in females (*t=*−2.84, d.f.=15, *P*=0.012).

The time to peak tetanus (*t_P_*_0_) was significantly different between sexes (*t=*–2.89, d.f.=15, *P*=0.011; male DTB: 38.90±16.00 ms, female DTB: 62.68±20.64 ms).

To assess whether commonly used isometric contractile parameters can predict *V*_max_, we assessed whether any of the measured properties correlated. Of the measured twitch parameters, we found both *t_P_*_tw_* *and FWHM negatively correlated with *V*_max_ (*t_P_*_tw_* *and *V*_max_ *r*= −0.41, *N*=17, *P*=0.045; FWHM and *V*_max_ *r*=−0.40, *N*=17, *P*=0.048), but RT_50_ did not correlate significantly with *V*_max_ (*r*=−0.21, *N*=17, *P*=0.20). Of the measured tetanic parameters, *t_P_*_0_ negatively correlated with *V*_max_ (*r*=−0.50, *N*=17, *P*=0.018). Faster twitches and tetani are thus associated with increased shortening speed (Fig. 5).

### Comparison of force–length methodologies

We tested whether a 1 s duration stimulation during cyclical motion provides similar force–length profiles as a stepwise methodology for syringeal muscles (Fig. 2). Stepwise methodologies gave mean *L*_0_ values of 5.16±0.23 and 4.98±0.23 mm in males (*N*=8) and females (*N*=6), respectively. Cyclic methodologies gave mean *L*_0_ values of 5.07±0.23 and 5.06±0.24 mm. Peak stresses at *L*_0_ were 4.45±0.64 mN mm^−2^ in males and 7.05±1.13 mN mm^−2^ in females using stepwise methodologies, whereas cyclic methodologies returned peak stresses of 3.60±0.72 mN mm^−2^ in males and 5.42±0.64 mN mm^−2^ in females (Fig. 2F). Pairwise comparisons revealed methodologies did not significantly differ in *L*_0_ (*t*=0.40, d.f.=13, *P*=0.70) or σ_0_ (*t*=0.25, d.f.=13, *P*=0.81). Thus, both methodologies result in the same values for *L*_0_ in both male and female preparations (Fig. 2E).

To test whether the prolonged stimulation (1 s) during cyclic approaches affected muscle function, we tested whether tetani 5 min after this differed from those before. We found no significant differences in male (before: 4.11±2.13 mN mm^−2^, after: 3.44±0.92 mN mm^−2^; *t=*0.54, d.f.=7, *P*=0.61) or female (before: 5.53±2.57 mN mm^−2^, after: 6.38±3.15 mN mm^−2^; *t*=−1.06, d.f.=5, *P*=0.34) DTB muscle (Fig. 2G). Thus, 1 s duration stimulation did not affect σ_0_.

## DISCUSSION

With a maximum shortening velocity of 25*L*_0_ s^−1^ at 30°C, and several individuals up to 31*L*_0_ s^−1^, zebra finch superfast syringeal muscle has one of the highest shortening velocities measured to date. Using a conservative estimate for *Q*_10_, we suggest that DTB muscle could achieve maximum shortening velocities of between 37 and 42*L*_0_ s^−1^ on average. Compared with established superfast muscles at similar temperatures, syringeal muscle achieves much greater shortening velocities than rattlesnake tail shaker muscle (18*L*_0_ s^−1^ at 35°C; Rome et al., 1996; Table 3), insect flight muscles (10.1–16.1*L*_0_ s^−1^ at 35°C; Table 3) and rabbit extraocular muscle, a potential superfast muscle (23*L*_0_ s^−1^ at 35°C; Briggs and Schachat, 2000; Table 3). We currently lack force–velocity data on toadfish swimbladder and bat laryngeal muscles at physiologically relevant temperatures (Elemans et al., 2011; Rome, 2006). Comparisons with other vocal muscles reveal considerably faster shortening in syringeal muscle. The laryngeal TA muscle of humans shortens between 2.55*L*_0_ s^−1^ at 12°C and 2.9*L*_0_ s^−1^ at 15°C, and that in rats ranges up to 13*L*_0_ s^−1^ at 25°C (Table 3). The high shortening velocities reported here in avian syringeal muscles are, to the best of our knowledge, only exceeded by estimated values for myotomal muscles in larval zebrafish that are used for fast C-starts in escape responses. These muscles have been estimated to achieve up to 45*L*_0_ s^−1^ at 28°C (Mead et al., 2020) on the assumption that workloop shortening represents 40% of the muscle *V*_max_. In general, the differences in maximal shortening velocity between muscles reflects their optimisation for different features and different functions. These differences are reflected in the fibre-type compositions of muscles (Adam et al., 2023; Hoh, 2005), suggesting further work may be required to isolate individual fibres and measure sarcomere shortening velocities directly (Seow and Ford, 1991; West et al., 2013). The high shortening velocities in syringeal muscles again highlights that they are tuned for speed.

**Table 3.**
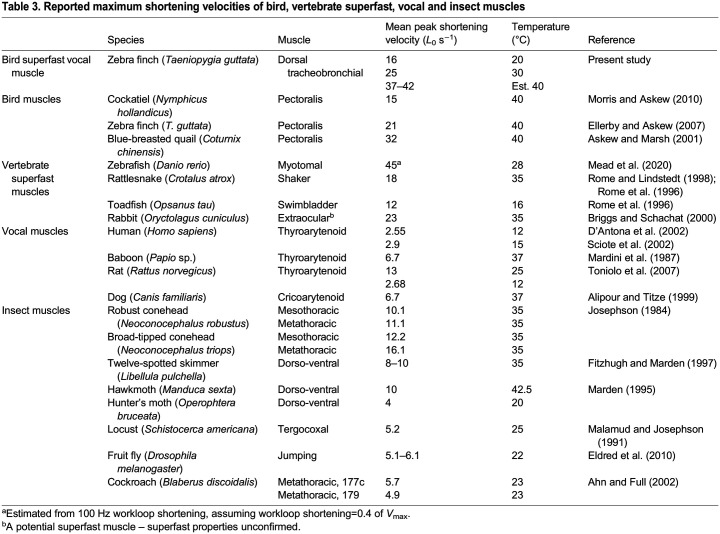
Reported maximum shortening velocities of bird, vertebrate superfast, vocal and insect muscles

In many of our muscle preparations, force did not reach steady-state during the isovelocity curve. This could be caused by the high passive stiffness and series elasticity of these muscles (Mead et al., 2017), and could possibly lead to an overestimation of *V*_max_. However, when using the ramp manoeuvre of Claflin and Faulkner (1989), which includes a length shortening step prior to the isovelocity ramp (protocol 2), our preparations were able to reach steady-state. As the step size required increases with release velocity, we found that the required step was too large (in excess of 5% of *L*_0_) to give sufficient time for the isovelocity manoeuvre at velocities over ∼17.5*L*_0_ s^−1^. We note other studies without the preceding step do also not achieve steady-state in faster muscles, such as in the EDL of mice (Barclay et al., 1993, 2010), larval zebrafish myotomal muscle (Mead et al., 2020) and frog (*Rana pipiens*) sartorius muscle (Seo et al., 1994). Because the maximum velocity estimates did not differ between either of the protocols tested, we conclude that our reported *V*_max_ values are not overestimated. However, when using whole muscle, or muscle fascicles, we cannot be sure that the sarcomere velocity is the same as the velocity imposed during the isovelocity manoeuvre. Fully understanding this, and to account for the noted high passive stiffness and series elasticity of syringeal muscles, requires further work assessing sarcomere velocity directly.

Our data show that maximal shortening velocities correlate positively with isometric properties, corroborating previous work (Close, 1965; Lännergren, 1978). Despite these correlations, and even though our isometric measurements confirm that male twitches are nearly twice as fast as female twitches (Adam and Elemans, 2020), superfast shortening was not significantly different between sexes. Although we highlight these correlations, we note that these represent a small sample size of 17 animals, which is likely underpowered and may be influenced more strongly by natural variability between preparations. Even so, disparities between twitch speed and shortening velocities have been shown in other muscles. For example, in cuttlefish (*Sepia officinalis*) mantle muscle, juveniles have significantly faster twitches than adults, but shortening velocities do not differ between the two age groups (Gladman and Askew, 2022). These disparities were suggested to result from twitch dynamics being more closely aligned with cyclic muscle performance than with shortening velocity (Gladman and Askew, 2022; Marsh, 1990). Twitch activation and relaxation times are determined by the deactivation of cross-bridge cycling. The high energetic costs associated with rapid deactivation mean that isometric contractile properties are usually tightly coupled with the operating frequency of muscle seen during movement and, in this case, song. This is achieved by maintaining a specific ratio between contraction kinetics and cycle duration, regardless of whether the muscle is activated through a single stimulus or through a burst of stimuli (Askew and Marsh, 2001; Gladman and Askew, 2022; Marsh, 1990). Girgenrath and Marsh (1999) emphasised the close link between muscle operating frequency and twitch duration across a range of taxa, and demonstrated that faster twitches were strongly associated with greater cycle frequencies. Thus, isometric data seem to more accurately predict *in vivo* cycling frequency than force–velocity parameters alone.

Previous attempts to predict the cyclic performance of muscle from isometric characteristics suggest that more accurate predictions require knowledge of force–length, force–velocity, activation and relaxation kinetics, as well as the roles of series elasticity and other non-contractile elements within tissues (Caiozzo, 2002; James et al., 1996; Josephson, 1993). James et al. (1996) were able to offer a good approximation of work loop performance of the latissimus dorsi muscle of Dutch rabbits by integrating both isometric and isotonic measures; however, the accuracy of these predictions declined with increased cycle frequency. More recent work has further developed these frameworks through the integration of sarcomere dynamics (Nguyen and Venkadesan, 2021 preprint), titin (Nishikawa, 2020; Whitney et al., 2022) and other elements (Ross et al., 2018; Wheatley et al., 2018). The increased complexity of these models enables more accurate predictions of work loop and/or *in vivo* performance, though the need for such complexity demonstrates the intricate interactions taking place within a muscle during work loop performance.

In tuning syringeal muscle to operate at high frequencies, we show that next to stress, instantaneous power has also been traded for speed. In zebra finches, the isometric stress of syringeal muscle is 7.13±4.85 mN mm^−2^ at 39°C (Adam et al., 2023), some 95–96% lower than that of the fast-twitch pectoralis at 40°C (167±26 mN mm^−2^; Ellerby and Askew, 2007). Reduced force-generating capacity results from decreased myofibrillar area (Mead et al., 2017), and cross-bridge duty cycles (Askew, 2023), which, together with high detachment rates, enable rapid shortening and cycling rates (Rome et al., 1999; Tikunov and Rome, 2009). The reduction in force-generating capacity is not adequately compensated for by increased speed, resulting in the instantaneous power of zebra finch syringeal muscle being substantially lower than that of pectoralis muscle (Ellerby and Askew, 2007). Instantaneous power is much greater than cyclic power. Elemans et al. (2008) reported the cyclic power of zebra finch syringeal muscle (the VTB muscle) reaches ∼6 W kg^−1^ at frequencies <100 Hz. At higher frequencies (200 Hz) this declines to ∼2 W kg^−1^. Disparities between cyclic and instantaneous power is a common feature, where cyclic power output is between 15 and 33% of instantaneous power in locomotory muscles (Ellerby and Askew, 2007; Gladman and Askew, 2022; James et al., 1996). Our estimates of power output at 40°C suggest that the maximum cyclic power output is 15–26% of the instantaneous power output. These similarities highlight that the underlying mechanical processes are likely similar between muscle types. Despite these underlying similarities, the power output of syringeal muscle is substantially reduced as a result of adaptations for high-speed operation.

Our measures of force–length parameters reveal that conventional stepwise approaches and continual stimulation during cyclic motion previously employed in laryngeal tissues (Alipour-Haghighi et al., 1991) yield the same *L*_0_ and stress. However, these cyclical measurements provide further detail about how the tissue responds during sinusoidal movements, and the role of passive and elastic elements during movement. These approaches, coupled with twitch activation and deactivation dynamics, may enable us to produce theoretical work loops. Despite this, the complex interactions suggest that such experiments would be best coupled with measures of work loops using the tissue itself at a similar temperature, enabling theoretical loops to be directly compared with how the tissue behaves *in vitro*. Indeed, during cyclic continual stimulation, we see passive stress is higher beyond *L*_0_ than seen using conventional stepwise approaches. This likely arises as the muscle is not reaching steady-state through the experimental procedure. Although either approach is appropriate for finding *L*_0_ in vocal muscles, the stepwise approach is likely less damaging, particularly in other muscles that may not show such high fatigue resistance. However, using cyclic force–length data may provide additional benefits in predicting work loop performance (Nguyen and Venkadesan, 2021 preprint), though further work is required to fully assess this.

Syringeal muscle showed a high degree of thermal dependence between 20 and 30°C. When compared with other potentially superfast muscles, we see similar levels of thermal dependence. The *Q*_10_ values of *V*_max_ in rabbit extraocular muscle and zebrafish myotomal muscle are 1.7 and 2.2, respectively (Asmussen et al., 1994; Mead et al., 2020), and the DTB muscle falls within this range (*V*_max_=1.74). Wider comparisons reveal that thermal dependence of rate-based processes is a common feature of muscle (see Bennett, 1984, 1985). Shortening velocity of locomotory muscle has a typical *Q*_10_ value of between 1.4 and 2.2. Other muscles that operate at high frequencies have similarly high thermal dependence: the twelve-spotted skimmer (*Libellula pulchella*) flight muscle *V*_max_ has a *Q*_10_ of approximately 2 between 18 and 28°C, falling to 1.13 between 28 and 38°C (Fitzhugh and Marden, 1997); the *V*_max_ of hawkmoth (*Manduca sexta*) flight muscle has a *Q*_10_ of 1.71 between 20 and 30°C and 1.67 between 30 and 40°C (Marden, 1995); and katydid flight muscle *V*_max_ *Q*_10_ ranges between 1.43 and 1.76 between 25 and 35°C (Josephson, 1984). These results show that the *Q*_10_ of DTB *V*_max_ falls within the range of previously reported values and that high-frequency muscles show a high degree of thermal dependence.

This high thermal dependence of superfast muscles suggests that muscle function is compromised at non-physiological temperatures. In other muscles that operate at high frequencies, such as the flight muscles of insects, high thermal dependence is also seen. In hawkmoths (*M. sexta*; Wilmsen and Dzialowski, 2023), bumblebees (*Bombus terrestris*; Masson et al., 2017) and variable field crickets (*Gryllus lineaticeps*; Sun et al., 2020), low temperatures (≤20°C) preclude flight, as flight muscle cannot generate the required work and power. At temperatures ≥25°C, bumblebees, crickets and hawkmoths perform a pre-flight warmup, a process similar to shivering; this increases the temperature of the thorax, enhancing flight muscle function (Masson et al., 2017; Wilmsen and Dzialowski, 2023). This process increases the body temperature rapidly; in crickets, a 5-min warm-up increases body temperature by 6°C (Sun et al., 2020). The flight muscle of many insects performs best at temperatures ≥35°C (Wilmsen and Dzialowski, 2023). High-frequency muscles of birds and bats are likely optimised to operate at similar body temperatures: when operating outside of these temperatures, we see greatly reduced force output, such as with the pectoralis muscle of hummingbirds, where drastic temperature reductions of ≤20°C produce substantially reduced force (Reiser et al., 2013). Even in endotherms, such significant reductions of body temperature are commonly seen and behaviourally relevant: small passerines reduce body temperature 5–10°C during the winter (Brodin et al., 2017; García-Díaz et al., 2023; Ruf and Geiser, 2015), and hummingbirds and bats reduce body temperature by ≤20°C during daily torpor to reduce energetic costs (Luo et al., 2021; Shankar et al., 2022). The underlying muscle mechanics may now affect behavioural performance. Indeed, Wu et al. (2021) found that the echolocation call frequency of leaf-nosed bats (*Hipposideros armiger*) decreased with decreased body temperature, and great tits (*Parus major*) switch from defending territories through elaborate song to using short, less demanding, alarm calls during cold days (Strauss et al., 2020). Thus, the thermal dependence in superfast vocal muscle may impact vocal behaviour.

## Supplementary Material

10.1242/jexbio.246330_sup1 Supplementary information
