# Supplementary material for: Male and female syringeal muscles exhibit superfast shortening velocities in zebra finches
Source: J Exp Biol. 2024 Apr 8;227(7):jeb246330. doi: 10.1242/jeb.246330 (PMC11058336; doi:10.1242/jeb.246330)
Supplement: Supplementary information [file jexbio-227-246330-s1.pdf]

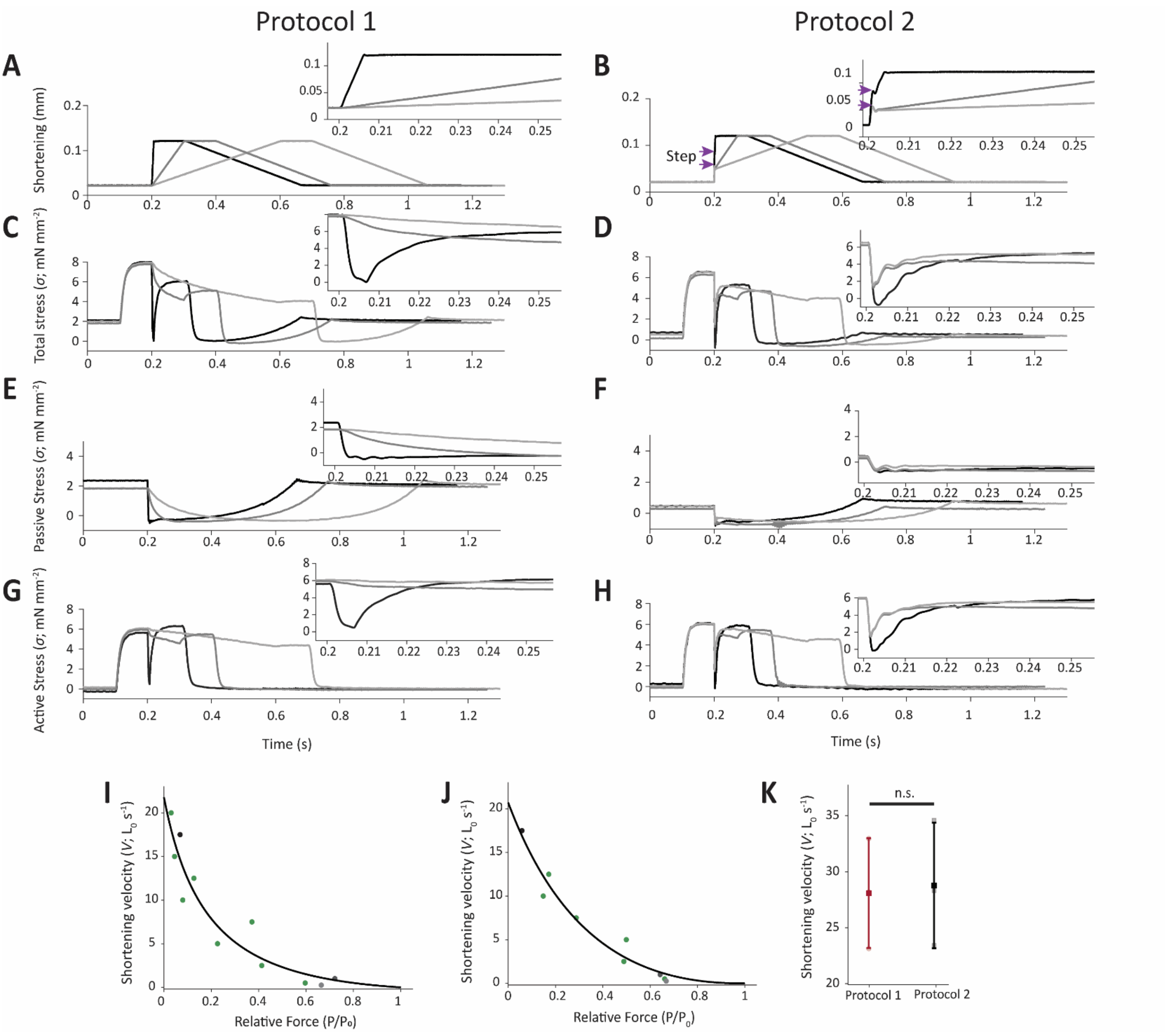

Fig. S1. Comparison of force-velocity methodologies. Force-velocity experiments based on protocol 1 (without preceding step) and protocol 2 (with a preceding step). Graded shades of grey are used to represent different ramp velocities in A-H. Data shown in A-J is from a left DTB preparation from one female animal (GW423) at 17.5 (black), 2.5 (dark grey) and 0.25 (light grey) L_0 s^{-1}. (A,B) Length changes at different ramp velocities (A) using protocol 1, and (B) protocol 2. (C,D) Resulting total stress output (C) using protocol 1 and (D) protocol 2. (E,F) Passive stress output (E) using protocol 1 and (F) protocol 2. (G, H) Active stress, calculated as (G) E-C (protocol 1) and (H) F-D (protocol 2). (I-J) Example force-velocity profiles from (I) protocol 1 and (J) protocol 2, curvature did not differ significantly between the two protocols (power ratios; t = 0.14, df = 2, p = 0.91, N = 3). (K) The two protocols did not return significantly different estimates of maximum shortening velocity (t = 0.84, df = 2, p = 0.49, N = 3).

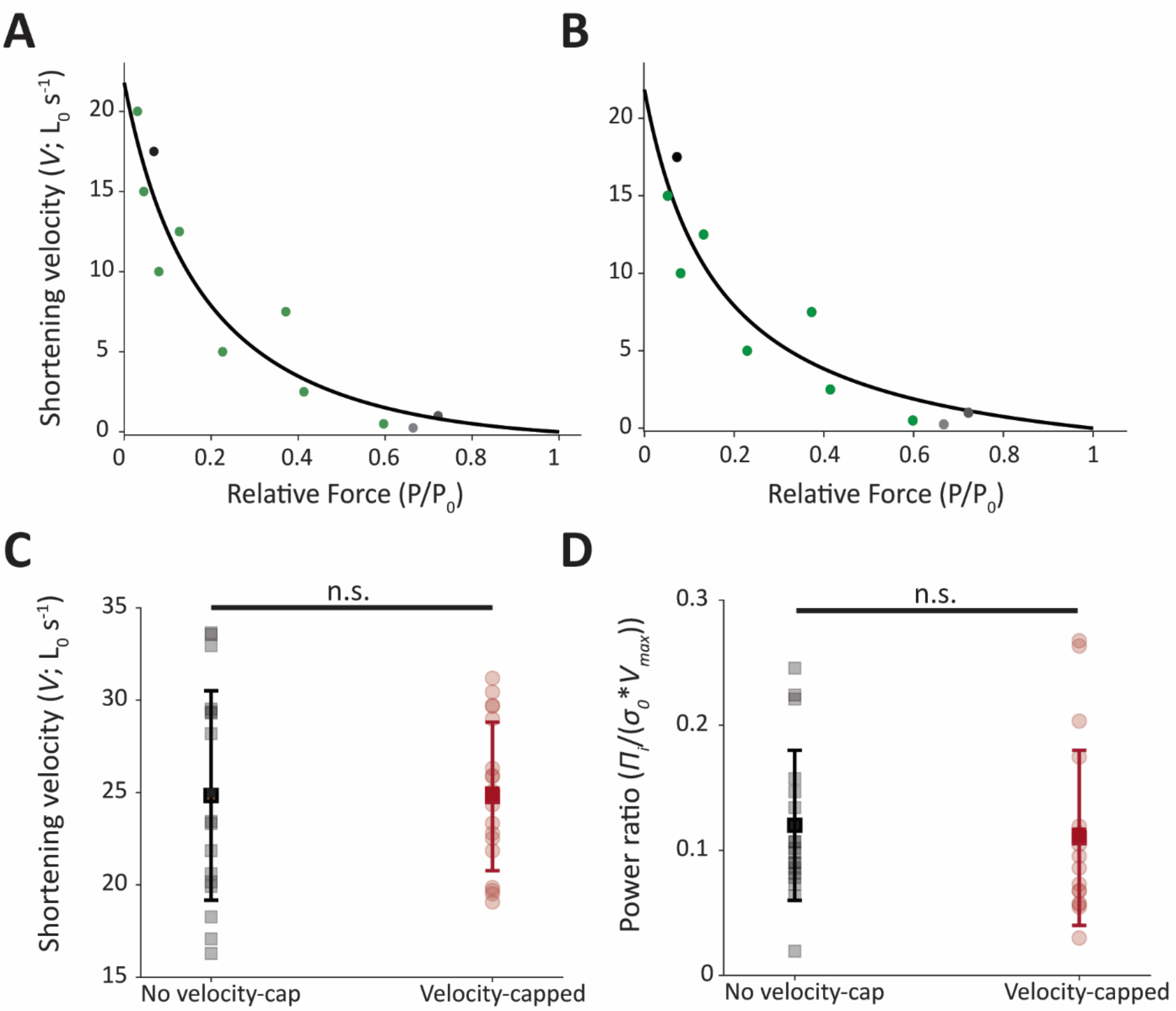

Fig. S2. Comparison of force-velocity profiles with and without a 17.5 L0 s^{-1} cap. (A) Example force-velocity profile without a velocity-cap. (B) Force-velocity profile from the same preparation with a 17.5 L0 s^{-1} cap. Use of a velocity-cap did not significantly change estimates of (C) *Vmax* (t = 0.06, df = 15, p = 0.95), or (D) curvature of the force-velocity relationship (power ratios; t = 0.32, df = 15, p = 0.75). Force-velocity profiles in (A) and (B) are both from a left DTB preparation from one female animal (GW423), graded grey points indicate data examples shown in Figure S1 at 17.5 (black), 2.5 (dark grey) and 0.25 (light grey) L0 s^{-1}.

Journal of Experimental Biology • Supplementary information

Table S1. Chemical composition of avian buffer solution. Solution was adjusted to pH 7.4 using Trizma at room temperature (approximately 21°C) prior to gassing with oxygen.

Chemical	Concentration (mM)
Sodium chloride (NaCl)	150
Potassium chloride (KCl)	2.5
Calcium chloride ($CaCl_2$)	4
Magnesium sulphate ($MgSO_4$)	1
Monosodium phosphate (NaH_2PO_4)	1
Hepes ($C_8H_{18}N_2O_4S$)	10
Glucose ($C_6H_{12}O_6$)	12
Trizma ($C_4H_{11}NO_3$)	1000

Journal of Experimental Biology • Supplementary information
